# A peripheral neutrophil-related inflammatory factor predicts a decline in executive function in mild Alzheimer’s disease

**DOI:** 10.1186/s12974-020-01750-3

**Published:** 2020-03-14

**Authors:** Kritleen K. Bawa, Saffire H. Krance, Nathan Herrmann, Hugo Cogo-Moreira, Michael Ouk, Di Yu, Che-Yuan Wu, Sandra E. Black, Krista L. Lanctôt, Walter Swardfager, for the Alzheimer’s Disease Neuroimaging Initiative

**Affiliations:** 1 Department of Pharmacology & Toxicology, University of Toronto, 1 King’s College Circle, Toronto, ON M5S 1A8 Canada; 2 Hurvitz Brain Sciences Program, Sunnybrook Research Institute, 2075 Bayview Avenue, Toronto, ON M4N 3M5 Canada; 3 Schulich School of Medicine & Dentistry, Western University, 1151 Richmond St, London, ON N6A 5C1 Canada; 4 Department of Psychiatry, Faculty of Medicine, University of Toronto, 250 College Street, 8th floor, Toronto, ON M5T 1R8 Canada; 5 Departamento Psichiatria, Universidade Federal de São Paulo, 1, R. Borges Lagoa, 570–Vila Clementino, Sao Paulo, SP 04038-000 Brazil; 6 Department of Education and Psychology: Division of Methods and Evaluation, Freie Universität Berlin, Fabeckstraße 37 & 69; Habelschwerdter Allee 45, 14195 Berlin, Germany; 7 Department of Neurology, Faculty of Medicine, University of Toronto, 6 Queen’s Park Crescent West, Third Floor, Toronto, ON M5S 3H2 Canada

**Keywords:** Neutrophil, Alzheimer’s disease, Executive function, Memory, Inflammation, Myeloperoxidase, Interleukin-8, Neutrophil gelatinase-associated Lipocalin, Tumor necrosis factor, Macrophage inflammatory protein-1 beta

## Abstract

**Background:**

Studies suggest a role of the innate immune system, including the activity of neutrophils, in neurodegeneration related to Alzheimer’s disease (AD), but prospective cognitive data remain lacking in humans. We aimed to investigate the predictive relationship between neutrophil-associated inflammatory proteins in peripheral blood and changes in memory and executive function over 1 year in patients with AD.

**Methods:**

Participants with AD were identified from the Alzheimer’s Disease Neuroimaging Initiative (ADNI). Neutrophil gelatinase-associated lipocalin (NGAL), myeloperoxidase (MPO), interleukin-8 (IL-8), macrophage inflammatory protein-1 beta (MIP-1β), and tumor necrosis factor (TNF) were assayed by luminex immunofluorescence multiplex assay at baseline. Confirmatory factor analysis was used to test an underlying neutrophil associated plasma inflammatory factor. Composite *z*-scores for memory and executive function were generated from multiple tests at baseline and at 1 year. A multiple linear regression model was used to investigate the association of the baseline inflammatory factor with changes in memory and executive function over 1 year.

**Results:**

Among AD patients (*n* = 109, age = 74.8 ± 8.1, 42% women, Mini Mental State Examination [MMSE] = 23.6 ± 1.9), the neutrophil-related inflammatory proteins NGAL (*λ* = 0.595, *p* < .001), MPO (*λ* = 0.575, *p* < .001), IL-8 (*λ* = 0.525, *p* < .001), MIP-1β (*λ* = 0.411, *p* = .008), and TNF (*λ* = 0.475, *p* < .001) were found to inform an underlying factor. Over 1 year, this inflammatory factor predicted a decline in executive function (*β* = − 0.152, *p* = 0.015) but not memory (*β* = 0.030, *p* = 0.577) in models controlling for demographics, brain atrophy, white matter hyperintensities, the ApoE ε4 allele, concomitant medications, and baseline cognitive performance.

**Conclusions:**

An inflammatory factor constructed from five neutrophil-related markers in peripheral blood predicted a decline in executive function over 1 year in people with mild AD.

## Introduction

Alzheimer’s disease (AD) is a complex neurodegenerative disorder characterized by the deposition of amyloid-β plaques and neurofibrillary tangles in the brain [1]. It is the most prevalent form of dementia seen in people over 65 years of age, and it causes severe deficits in memory and executive function. It is well established that patients with AD have elevated immune activation, which can be indicated by inflammatory markers in peripheral blood; however, studies have shown inconsistent results [2, 3]. This may be due in part to heterogeneity in immune responses that contribute or in the different markers examined between studies. Hence, it is important to identify particular inflammatory pathways that contribute to particular symptoms of the disease.

Recent studies suggest a role of innate immune activity, including the effects of neutrophils, in neurodegeneration related to AD [4, 5]. Neutrophils are involved in inflammation, pathogen clearance via phagocytosis, generation of reactive oxygen species (ROS) via myeloperoxidase activity, and neutrophil extracellular traps (NETs) [4]. Neutrophil activation and related oxidative stress have been associated with AD pathology in humans [5–7]. Evidence from clinical and animal studies suggests that neutrophils may translocate to and co-localize with the cerebral blood vessels and amyloid plaques within the brain parenchyma [8–11]. Recent animal studies have also shown that neutrophil adhesion in the cerebral small vessels may mediate changes in cognition [12, 13].

In a large study of 241 AD cases vs. 175 elderly controls with normal cognition, the neutrophil to lymphocyte ratio was significantly elevated in the AD cases [14]. Similar elevations in AD and mild cognitive impairment (MCI) groups were found in other studies [15, 16], such as the Australian Imaging and Biomarkers and Lifestyle study, where small correlations were also noted with amyloid burden and cognition [16]. While that study did not observe a statistically significant increased risk of developing AD, it did not assess the relationships between neutrophils and changes in sensitive cognitive measures over time in people with AD. Recently, Dong et al. reported correlations between different neutrophil activity parameters with change in MMSE score [6], while another study showed associations between expression of CD11b, an adhesion molecule found on neutrophils, with measures of mental impairment, and disease progression measured by the Token Test, but not with disease progression measured by the MMSE, in patients with AD [17].

The present study examines five neutrophil-related plasma markers in peripheral blood: myeloperoxidase (MPO) which is responsible for neutrophil-related oxidative stress [4, 18], neutrophil gelatinase-associated lipocalin (NGAL) a neutrophil secreted anti-microbial molecule [4, 19], tumor necrosis factor (TNF) which is involved in neutrophil activation and survival [20, 21], and interleukin-8 (IL-8) and macrophage inflammatory protein-1β (MIP-1β), which both play a role in neutrophil trafficking and activation and which are both secreted by activated neutrophils [22].

Because prospective cognitive data remain scant in human subjects, here, we investigate the longitudinal relationship between neutrophil-associated inflammatory proteins in peripheral blood plasma and changes in memory and executive function over 1 year in patients with AD. We hypothesized an inflammatory factor constructed from the five neutrophil-related markers would predict a decline in both memory and executive function.

## Methods

### Study population

The Alzheimer’s Disease Neuroimaging Initiative (ADNI) (http://adni.loni.usc.edu/) is a non-randomized, longitudinal observational study dedicated to recording the detection and progression of AD and associated biomarkers in North America. ADNI was conducted according to Good Clinical Practice guidelines, US 21CFR Part 50–Protection of Human Subjects, and Part 56–Institutional Review Boards, and pursuant to state and federal regulations. HIPAA authorizations and written informed consent were obtained from all study participants and/or authorized representatives and study partners.

In this analysis, participants of the first phase of ADNI, ADNI 1 (2004–2009) with mild AD were included. ADNI 1 recruited participants with mild AD based on pre-specified inclusion/exclusion criteria: memory complaint themselves or by their study partner, impaired memory function as measured by a score lower than the adjusted cutoff based on educational level on the Logical Memory II subscale (Delayed Paragraph Recall) from the Wechsler Memory Scale, a Mini Mental State Examination (MMSE) score between 20 and 26, Clinical Dementia Rating (CDR) of 0.5 or 1, and National Institute of Neurological and Communicative Disorders and Stroke and the Alzheimer’s Disease and Related Disorders Association (NINCDS/ADRDA) criteria for probable AD [23, 24]. More information about the clinical characteristics of ADNI participants with AD can be found elsewhere [23].

### Plasma inflammatory markers

Plasma biomarker concentrations were examined in a subset of ADNI 1 participants (http://adni.loni.usc.edu/, accessed on 10/02/2019). Blood draws were performed in the morning after overnight fasting, and plasma was collected and allowed to freeze on dry ice. The samples were sent for analysis on the same day as collection. Luminex immunofluorescence multiplex assays were used to quantify markers of interest (NGAL, MPO, MIP-1β, IL-8, and TNF) following a standardized protocol [25]. Quality control analyses were performed, and individual analytes were Box-Cox transformed for normality.

### Memory and executive function composite sores

Composite scores for memory (ADNI-MEM) [26] and executive function (ADNI-EF) [27] were generated by confirmatory factor analysis at baseline and month 12 as described previously (accessed on 12/06/2019). The ADNI-EF was created using baseline measures of Category Fluency-animals, Category Fluency-vegetables, Trails A and B, Digit span backwards, WAIS-R Digit Symbol Substitution, and 5 Clock Drawing items (circle, symbol, numbers, hands, time). The ADNI-MEM composite included measures from the RAVLT, ADAS-cog, Logical memory, and MMSE. The models were created using Mplus version 5 with theta parameterization and the WLSMV estimator. Model fit indices such as Confirmatory Fit Index (CFI) > 0.95, the Tucker Lewis Index (TLI) > 0.95 and the root mean squared error of approximation (RMSEA) < 0.05 were used to indicate good fit. The scores were scaled to the *Z* distribution (mean = 0 and SD = 1).

### Potential confounders

Other measures used in this study include age, sex, use of an immune-related medication (non-steroidal anti-inflammatory and corticosteroid medications), use of anti-dementia medications, specifically acetylcholinesterase inhibitors and memantine, ApoE genotype (number of the ε4 alleles), Mini Mental State Exam (MMSE) scores, and brain volumetric measures as described previously [28] (accessed January 2019). Briefly, FreeSurfer (version 4.3) was used to process brain volumes, including left and right hippocampal volumes, whole brain volume, and intracranial volume obtained by T1-weighted 1.5 T MRI and white matter hyperintensity volumes (WMH) extracted using run time proton density (PD), T1- and T2-weighted MRI input images, and fluid-attenuated inversion recovery (FLAIR) training images. Cross-sectional processing allowed between-subjects comparisons by segmenting each image according to a FreeSurfer defined atlas. Image quality control was performed at a single site and included inspection for quality of the image, compliance with protocol, and important medical findings [28].

### Statistical analyses

#### Confirmatory factor analysis

Confirmatory factor analysis (CFA) was used to estimate a single latent factor from multiple markers related to neutrophil activation [29]. Concentrations of five neutrophil-associated inflammatory proteins measured in plasma of patients with AD at their ADNI 1 baseline visit were used. The five neutrophil-associated proteins included were Neutrophil gelatinase-associated lipocalin (NGAL)/Lipocalin 2, myeloperoxidase (MPO), macrophage inflammatory protein-1β (MIP-1β)/CCL4, interleukin-8 (IL-8), and tumor necrosis factor (TNF). The model fit indices used to indicate acceptable model fit were CFI > 0.95, the TLI > 0.90, the RMSEA estimate < 0.05, the RMSEA probability > 0.05, standardized root mean square residual (SRMR) < 0.08, and chi-square *p* value > 0.05. CFA was performed in Mplusv8 [30].

#### Multiple linear regression modeling

Multiple linear regression models were used to determine the association between the neutrophil-related inflammatory factor at baseline and ADNI-EF and ADNI-MEM, 12 months later. Significance was corrected considering two comparisons (*p* < .025). The models included ten covariates considered to be potential confounders a priori (age, gender, number of ApoE ε4 alleles, the baseline measure of ADNI-EF or ADNI-MEM, use of immune-related medications over the study period, use of acetylcholinesterase inhibitors or memantine over the study period, baseline white matter hyperintensity volume, and brain atrophy). White matter hyperintensity volumes were log base 10 transformed to produce normality. Brain atrophy was inferred using brain parenchymal fraction calculated by dividing whole brain volume by intracranial volume. Post hoc models included years of education and mean hippocampal volume [left hippocampal volume + right hippocampal volume divided by 2] as potential confounders. Missing data were imputed using maximum likelihood estimation (MLR) with robust standard errors and restriction of 0.05 minimum covariance coverage. The model fit was assessed using the same indices as above.

## Results

### Participant characteristics

Of 819 participants, 188 had a diagnosis of mild AD, and plasma biomarkers were available for 112. After excluding participants who had missing values for baseline characteristics (1 for white matter hyperintensity volume, and 2 participants had missing values for baseline whole brain volume, which was used to calculate brain atrophy), 109 participants were included. Baseline characteristics of the included participants are presented in Table 1.

**Table 1:** Baseline characteristics of mild AD patients (*n* = 109)

Screening/baseline demographics	Mean ± SD *or* median [IQR]	Association with neutrophil factor (standardized *β*)	*p* value
Age (years)	74.8 ± 8.1	0.292	0.011
Male	58%	− 0.139	0.572
Education (years)	15.1 ± 3.2	− 0.052	0.670
ApoE ε4 allele (number)	“0” = 31% “1” = 49% “2” = 20%	− 0.194	0.125
MMSE score	23.6 ± 1.9	− 0.118	0.351
CDR	0.74 ± 0.25	− 0.022	0.871
Brain parenchymal fraction	0.62 ± 0.04	− 0.064	0.644
White matter hyperintensity volume (cm^3^)	0.36 [0.12, 0.91]	− 0.071	0.598
Hippocampal volume (cm^3^)	5.72 ± 2.71 (*n* = 81)	− 0.209	0.195
Anti-inflammatory medication use (NSAID and corticosteroids use)	54%	0.014	0.956
Corticosteroid use	5%	− 0.283	0.367
NSAID (excluding ASA 81 mg) use	25%	− 0.400	0.177
ASA (81 mg) use	35%	0.364	0.141
Acetylcholinesterase inhibitor use	88%	− 0.390	0.390
Memantine use	45%	0.489	0.029

### Characteristics of the neutrophil-related inflammatory factor

**Fig. 1:**
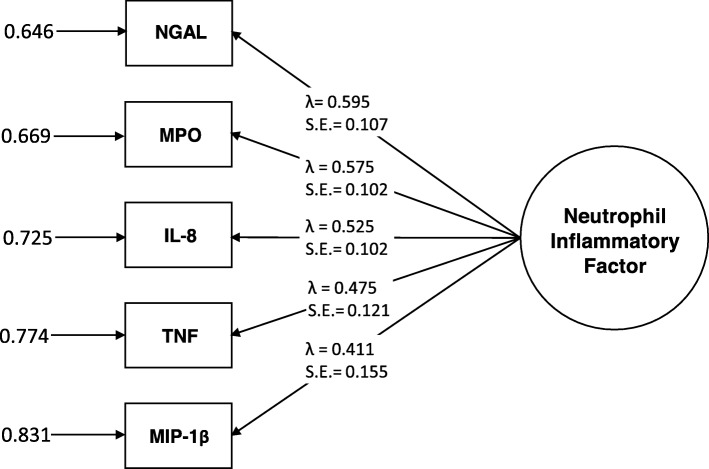
Peripheral inflammatory factor composed of neutrophil-related inflammatory protein plasma concentrations. Residual variances at left, factor loadings (*λ*), and standard errors (S.E.) center. MPO myeloperoxidase, TNF tumor necrosis factor, NGAL neutrophil gelatinase-associated lipocalin, MIP-1β macrophage inflammatory protein-1β, IL-8 interleukin-8

A neutrophil-related inflammatory factor estimated from plasma concentrations of NGAL, MPO, MIP-1β, IL-8, and TNF in AD patients (*n* = 109) showed excellent model fit as indicated by above adequate measures of all fit indices (CFI = 1.000, TLI = 1.068, RMSEA estimate = 0.000, RMSEA probability = 0.690, SRMR = 0.038, chi-squared *p* value = 0.5557). This suggested that the five measures adequately informed an underlying construct. The factor loadings, residual variances, and *p* values are shown in Fig. 1. Associations between the neutrophil-related inflammatory factor and participant characteristics are shown in Table 1.

### Participant outcomes

At baseline, participants’ mean ADNI-EF score was − 0.98 ± 0.90 *z*-score units, and this was not associated with the neutrophil inflammatory factor (standardized estimate [*β*] = 0.060, *p* = 0.723). Between baseline and 12-month follow-up (*n* = 96 complete cases), executive function declined to − 1.28 ± 0.99 *z*-score units (*t*_1,95_ = 5.471, *p* < .001). At baseline, participants’ mean memory score was − 0.83 ± 0.54 *z*-score units, and this was not associated with the baseline neutrophil inflammatory factor (*β* = 0.096, *p* = 0.612). Over 12 months, memory performance declined to − 1.04 ± 0.63 *z*-score units (*t*_1,95_ = 5.938, *p* < .001).

### Longitudinal associations between the neutrophil-related inflammatory factor and cognition

**Fig. 2:**
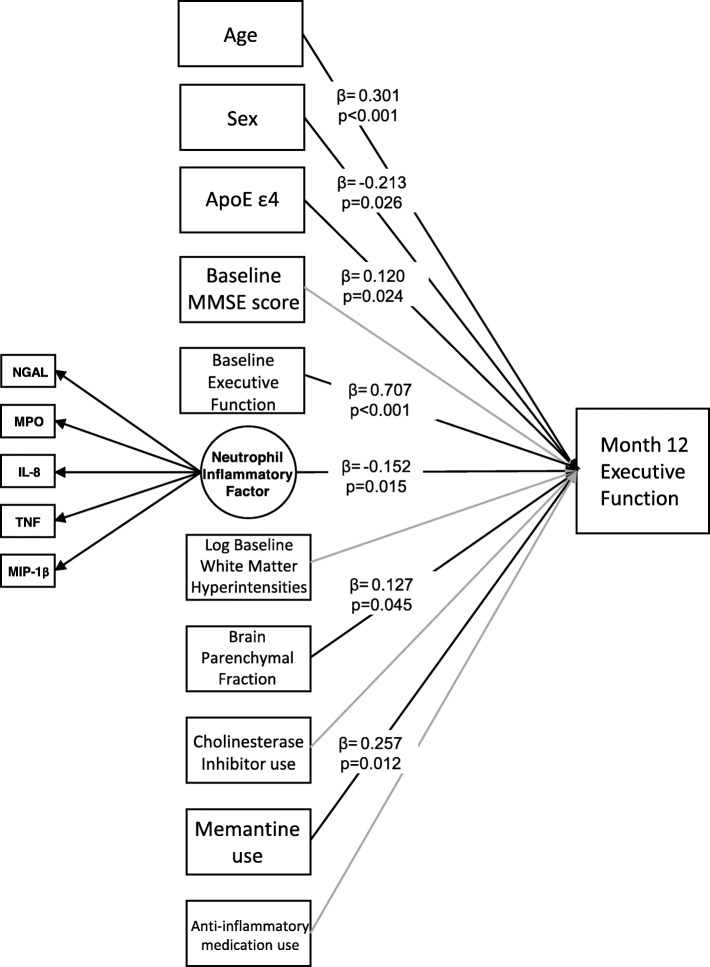
Linear regression model predicting executive function *z*-scores at month 12 controlling for covariates and baseline executive function *z*-scores

The inflammatory factor at baseline significantly predicted a decline in executive function at month 12 (*β* = − 0.152, *p* = 0.015; Fig. 2) in the adjusted model. The fit indices indicated a good model fit (CFI = 1.000, TLI = 1.033, RMSEA estimate = 0.000, RMSEA probability = 0.947, SRMR = 0.078, chi-squared *p* = 0.6402). Of the 10 covariates, age (standardized estimate (*β*) = 0.301, *p* < 0.001), sex (*β* = − 0.213, *p* = 0.026), baseline executive function (*β* = 0.707, *p* < 0.001), number of ApoE ε4 alleles (*β* = 0.120, *p* = 0.024), use of memantine (*β* = 0.257, *p* = 0.012), and baseline brain parenchymal fraction (*β* = 0.127, *p* = 0.045) were significantly associated with executive function at month 12. The 95% confidence intervals for the standardized estimates (*β*) in this model are provided in Supplementary Table 1.

**Fig. 3:**
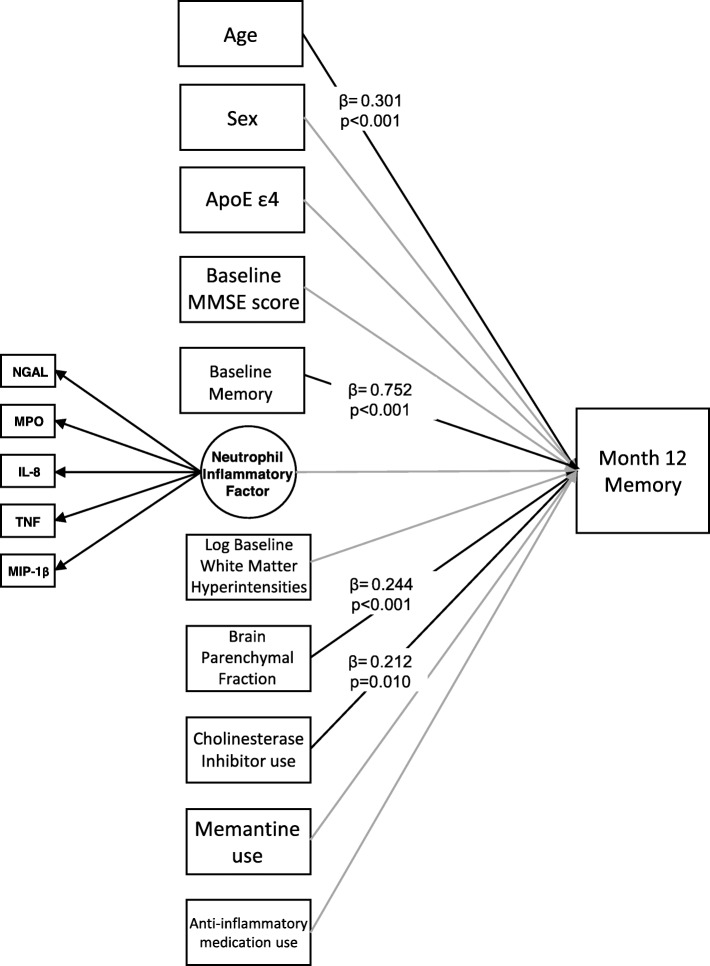
Linear regression model predicting memory *z*-scores at month 12 controlling for covariates and baseline memory *z*-scores

The inflammatory factor at baseline was not significantly associated with memory at month 12 (*β* = 0.030, *p* = 0.577; Fig. 3) in the adjusted model. The fit indices informed good model fit (CFI = 1.000, TLI = 1.047, RMSEA estimate = 0.000, RMSEA probability = 0.966, SRMR = 0.074, chi-squared *p* value = 0.7178). Of the 10 covariates, age (*β* = 0.301, *p* < 0.001), baseline memory score (*β* = 0.752, *p* value< 0.001), use of acetylcholinesterase inhibitor use (*β* = 0.212, *p* = 0.010), and baseline brain atrophy (*β* = 0.244, *p* < 0.001) were significantly associated with memory at month 12. The 95% confidence intervals for the standardized estimates (*β*) in this model are provided in Supplementary Table 2.

### Post hoc models

In post hoc models controlling for alternative potential confounders (e.g., hippocampal volume and years of education, acetylsalicylic acid [ASA] 81 mg), the relationship between the neutrophil-related inflammatory factor and executive function persisted, and it was of similar effect size (see Table 2). To ensure that the results were not due to the previously described relationship between cognitive decline and TNF, a further post hoc model was constructed removing TNF from the inflammatory factor. In a multiple linear regression model (Model 6, Table 2), the 4-marker inflammatory factor without TNF significantly predicted a decline in ADNI-EF at month 12 (*β* = − 0.137, *p* = 0.039).

**Table 2:** Post hoc models

Covariates	Model 1 (Fig. 2)	Model 2	Model 3	Model 4	Model 5	Model 6
Age	X	X	X	X	X	X
Sex	X	X	X	X	X	X
ApoE ε4 allele (number)	X	X	X	X	X	X
Years of education		X				
Baseline MMSE	X		X	X	X	X
Baseline EF	X	X	X	X	X	X
Baseline brain parenchymal volume (cross-sectional)	X		X	X	X	X
Baseline white matter hyperintensity volume	X	X	X	X	X	X
Baseline hippocampal volume (cross-sectional)		X				
Acetylcholinesterase inhibitor use	X	X	X	X	X	X
Memantine use	X	X	X	X	X	X
Anti-inflammatory (corticosteroid, NSAID, and ASA 81 mg)	X	X				X
Corticosteroid use			X			
NSAID use				X		
ASA 81 mg use					X	
Markers included in model	NGAL, MPO, IL-8, MIP-1β, TNF	NGAL, MPO, IL-8, MIP-1β, TNF	NGAL, MPO, IL-8, MIP-1β, TNF	NGAL, MPO, IL-8, MIP-1β, TNF	NGAL, MPO, IL-8, MIP-1β, TNF	NGAL, MPO, IL-8, MIP-1β
Model fit indices	X^2^*p* value 0.6402, RMSEA 0.000, RMSEA *p* value 0.947, CFI 1.000, TLI 1.033, SRMR 0.078	X^2^*p* value 0.5295, RMSEA 0.000, RMSEA *p* value 0.854, CFI 1.000, TLI 1.012 SRMR 0.084	X^2^*p* value 0.5819, RMSEA 0.000, RMSEA *p* value 0.930, CFI 1.000, TLI 1.012, SRMR 0.078	X^2^*p* value 0.5088, RMSEA 0.000, RMSEA *p* value 0.904, CFI 1.000, TLI 1.006, SRMR 0.079	X^2^*p* value 0.6964, RMSEA 0.000, RMSEA *p* value 0.961, CFI 1.000, TLI 1.045, SRMR 0.079	X^2^*p* value 0.6774, RMSEA 0.000, RMSEA *p* value 0.940, CFI 1.000, TLI 1.042, SRMR 0.073
Sample size	109	81	109	109	109	109
Effect of baseline neutrophil factor on follow-up executive function (standardized *β* [95% confidence interval], *p* value)	*β* = − 0.152 [− 0.274, − 0.029], *p* = 0.015	*β* = − 0.146 [− 0.288, − 0.003], *p* = 0.045	*β* = − 0.134 [− 0.273, − 0.011], *p* = 0.033	*β* = − 0.131 [− 0.252, − 0.009], *p* = 0.036	*β* = − 0.138 [− 0.252, − 0.009], *p* = 0.026	*β* = − 0.137 [− 0.267, − 0.007], *p* = 0.039

## Discussion

The present results suggest that markers collectively related to neutrophil activation predicted a small decline in executive function, but not in memory, in patients with mild AD. The results add to a previous longitudinal study that reported weak correlations between the neutrophil-to-lymphocyte ratio and cortical amyloid and weak correlations between the neutrophil-to-lymphocyte ratio and composite memory and non-memory cognitive scores [16]. Though executive dysfunction has been studied less commonly than memory in AD, it contributes to a decline in activities of daily living [31] and quality of life for AD patients [32]; therefore, predictors of executive function decline are clinically important. The present study used CFA to concatenate the variances in five inflammatory proteins related to neutrophil activation. The five markers returned a model with good fit, providing evidence that they can be considered to inform an underlying construct.

The marker with the highest factor loading on the latent variable was NGAL, also known as lipocalin-2. NGAL is a pro-inflammatory molecule selectively secreted in neutrophil granules [4]. It is secreted into peripheral circulation by neutrophils and endothelial cells, and it prevents the growth of bacterial colonies [19, 33]. Human postmortem studies have shown increased levels of NGAL in brain areas affected by AD [34, 35]. Moreover, patients with AD or MCI have increased levels of plasma NGAL as compared with controls, and it has been correlated with cognitive decline [36]. Our study provides evidence that NGAL is part of an inflammatory response involved in cognitive decline in AD, particularly a decline in executive function.

MPO, an enzyme found mainly in the azurophilic granules of neutrophils, produces reactive oxygen species upon neutrophil degranulation [4, 18]. It has been found in increased concentrations in the plasma of patients with AD [37] and elevated levels of both MPO, and oxidative products have been reported in the cortex and hippocampus in AD brains ( [38]). Plasma concentrations of MPO have been found to correlate with plasma concentrations of the Aβ_1–42_ peptide ( [37]), and increased MPO immunoreactivity has been found in both neurofibrillary tangles and amyloid plaques in AD brain tissue [38]. Polymorphisms in the promoter region of the MPO gene have also been linked to risk of cognitive decline [39] and AD [40] in elderly populations, but different studies have reported inconsistent results. Previous studies in mouse models established that neutrophil-derived MPO can promote blood brain barrier dysfunction and endothelial damage during inflammation [41]. A recent study showed that an MPO-deficient mouse model of AD was protected against cognitive decline [42]. Consistent with the previous results, our study may indicate an association between neutrophil adherence and degranulation with executive decline in AD [43, 44].

Interleukin-8 is a chemokine involved in the recruitment and activation of neutrophils in response to injury or infection [22, 45]. Additionally, neutrophils can secrete IL-8 when activated by certain pathogens, which amplifies neutrophil recruitment to the site of infection [22]. Previous studies have shown an increase in the concentrations of peripheral IL-8 in patients with AD versus controls [46], and a recent meta-analysis linked an IL-8 gene polymorphism with AD risk in populations of different ethnicities [47]. IL-8 release has also been implicated in neurotoxicity and neuronal cell death in vitro [48], and an IL-8 receptor antagonist showed neuroprotective benefits in a mouse model of AD [49]. A recent study showed that serum IL-8 concentrations were associated with WMH on T2-weighted MRI in patients with AD [50]. The present study adds evidence that peripheral IL-8 concentrations in AD might partly indicate neutrophil chemoattractant and activator functions, which predict a decline in executive function.

Although not a specific marker of neutrophil activation, TNF is a potent promoter of neutrophil activation, and it is involved in neutrophil infiltration, degranulation, and survival during inflammation [20, 21, 51]. Previous evidence suggests that peripheral TNF, either related to acute or chronic inflammatory processes, can predict cognitive decline in AD [52]. Here, we offer some evidence that TNF can be part of an inflammatory response related to multiple neutrophil inflammatory markers, which predicts a decline in executive function in mild AD. Excluding TNF from the models yielded similar findings, suggesting that the cognitive effects were related to the common variance in neutrophil-related markers, and not just to TNF per se.

Also known as CCL4, MIP-1β is a chemokine involved in neutrophil trafficking to the tissues, and it is secreted by neutrophils following their adhesion to the basement membrane of blood vessels [22, 53]. Although MIP-1β is not generally detected in the brains of healthy humans, a study reported a significant increase in the concentrations of MIP-1β in the brains of AD mouse models, positively correlated with amyloid deposition [54]. In a postmortem study, MIP-1β secreting astrocytes were also more abundant in the brains of AD patients versus controls [55]. MIP-1β positive astrocytes were found to be associated with amyloid deposits, and they were usually localized in the hippocampal formation and the entorhinal cortex [55]. MIP-1β can also be secreted by human brain microvascular endothelial cells, suggesting a role of this chemokine in promoting leukocyte extravasation and infiltration into the brain [56]. This would be consistent with the suggestion that neutrophils can adhere to cortical blood vessels, adding to microvascular inflammation and changes in cognition in the mouse [12, 13].

Deficits in executive function can be indicative of vascular contributions to cognitive impairment, which are often related to WMH [57]. As a possible limitation, the participants from ADNI 1 studied here had generally low volumes of WMH, and more subtle measures of vascular dysfunction (e.g. white matter microstructural damage or cerebral blood flow) were not ascertained, precluding evaluation of more subtle vascular measures. Future studies might examine relationships between neutrophil markers and vascular brain changes [58] either not present or not measured in ADNI 1 (e.g. WMH on FLAIR imaging, fractional anisotropy by diffusion tensor imaging, or cerebral blood flow by arterial spin labelling) as potential mediators of the relationship between neutrophil markers and executive decline. Zlokovic et al. suggest that vascular injury and blood brain barrier disruption are associated with early cognitive decline independent of amyloid and tau [43]. As a further limitation to the present study, amyloid and tau imaging were unavailable in these participants, precluding investigation as correlates of the inflammatory factor identified, or as criteria to detect important heterogeneity factors in the etiologies of the dementia cases studied. Because ADNI did not study dementia due to other neurodegenerative diseases, further work would be needed to determine if the observed associations are specific to AD. The sample size assessed was relatively small, with follow-up data in only 96 patients. Although neutrophil counts were not provided by ADNI, the current findings using neutrophil-related markers add to case-control [14] and prospective [16] studies that examined neutrophil counts. Finally, some of neutrophil-related markers can originate from the kidneys [59, 60]. Since renal dysfunction has previously been linked to higher risk of developing dementia [61], future studies with larger samples might investigate kidney disease or glomerular filtration rate as possible confounders. The relatively small sample size also precluded examining subgroups of men and women, limiting comment on the generalizability of the findings to men and women specifically.

## Conclusions

Our findings suggest the potential utility of investigating distinct inflammatory pathways affecting one or more particular symptoms of the disease. Inflammation has been suggested to affect mood [62, 63], memory, and other cognitive domains in AD [2, 3], but inconsistent results have prevented the translation of this research into clinical diagnostic/prognostic biomarkers or drug targets. Examining a subset of inflammatory markers belonging to a particular biological cascade, or to a type of inflammatory cell, might provide unique insights into the inflammatory signatures that are related to particular symptoms.

Using a latent factor composed of peripheral neutrophil-associated inflammatory proteins, the present data suggest a possible contribution of neutrophil adhesion and activation to the decline in executive function over time in mild AD. Future studies might examine relationships of neutrophil markers with amyloid, tau, and indicators of cerebrovascular disease.

## Supplementary information

**Additional file 1: Supplementary Table 1.** 95% Confidence Intervals for standardized estimates (β) in model predicting change in executive function over one year

**Additional file 2: Supplementary Table 2.** 95% Confidence Intervals for standardized estimates (β) in model predicting change in memory over one year

## Data Availability

Data used in preparation of this article were obtained from the Alzheimer’s Disease Neuroimaging Initiative (ADNI) database (adni.loni.usc.edu). As such, the investigators within the ADNI contributed to the design and implementation of ADNI and/or provided data but did not participate in analysis or writing of this report.

## Abbreviations

AD: Alzheimer’s disease
ADAS-cog: The Alzheimer’s Disease Assessment Scale–Cognitive test
ADNI: Alzheimer’s Disease Neuroimaging Initiative
ADNI-EF: Composite scores for executive function
ADNI-MEM: Composite scores for memory
ApoE4: Apolipoprotein E4
CDR: Clinical Dementia Rating
CFA: Confirmatory factor analysis
CFI: Confirmatory Fit Index
FLAIR: Fluid-attenuated inversion recovery
GFR: Glomerular filtration rate
IL-8: Interleukin-8
MCI: Mild cognitive impairment
MIP-1β: Macrophage inflammatory protein-1 beta
MLR: Maximum likelihood estimation
MMSE: Mini Mental State Examination
MPO: Myeloperoxidase
NETs: Neutrophil extracellular traps
NGAL: Neutrophil gelatinase-associated lipocalin
NINCDS/ADRDA: National Institute of Neurological and Communicative Disorders and Stroke and the Alzheimer’s Disease and Related Disorders Association
RAVLT: Rey Auditory Verbal Learning Test
RMSEA: Root mean squared error of approximation
ROS: Reactive oxygen species
SRMR: Standardized root mean square residual
TLI: Tucker Lewis Index
TNF: Tumor necrosis factor
WLSMV estimator: Weighted least square mean and variance adjusted estimator
WMH: White matter hyperintensity volumes
