# Supplementary material for: A peripheral neutrophil-related inflammatory factor predicts a decline in executive function in mild Alzheimer’s disease
Source: J Neuroinflammation. 2020 Mar 14;17:84. doi: 10.1186/s12974-020-01750-3 (PMC7071641; doi:10.1186/s12974-020-01750-3)
Supplement: Supplementary file 1 — Additional file 1: Supplementary Table 1. 95% Confidence Intervals for standardized estimates (β) in model predicting change in executive function over one year [file 12974_2020_1750_MOESM1_ESM.docx]

**Supplementary Table 1: 95% Confidence Intervals for standardized estimates (β) in model predicting change in executive function over one year**

| **Predictors of follow-up executive function** | **2.5%** | **97.5%** | **β** |
| --- | --- | --- | --- |
| Neutrophil-related Inflammatory Factor | -0.274 | -0.029 | -0.152 |
| Age | 0.180 | 0.422 | 0.301 |
| Sex | -0.401 | -0.025 | -0.213 |
| ApoE ε4 | 0.016 | 0.223 | 0.120 |
| Baseline MMSE score | -0.135 | 0.095 | -0.020 |
| Baseline Executive Function | 0.597 | 0.816 | 0.707 |
| Log Baseline White Matter Hyperintensities | -0.136 | 0.053 | -0.042 |
| Brain Parenchymal Fraction | 0.003 | 0.251 | 0.127 |
| Cholinesterase Inhibitor use | -0.457 | 0.398 | -0.030 |
| Memantine use | 0.057 | 0.456 | 0.257 |
| Anti-inflammatory medication use | -0.058 | 0.347 | 0.144 |
