# Supplementary material for: A peripheral neutrophil-related inflammatory factor predicts a decline in executive function in mild Alzheimer’s disease
Source: J Neuroinflammation. 2020 Mar 14;17:84. doi: 10.1186/s12974-020-01750-3 (PMC7071641; doi:10.1186/s12974-020-01750-3)
Supplement: Supplementary file 2 — Additional file 2: Supplementary Table 2. 95% Confidence Intervals for standardized estimates (β) in model predicting change in memory over one year [file 12974_2020_1750_MOESM2_ESM.docx]

**Supplementary Table 2: 95% Confidence Intervals for standardized estimates (β) in model predicting change in memory over one year**

| **Predictors of follow-up memory** | **2.5%** | **97.5%** | **β** |
| --- | --- | --- | --- |
| Neutrophil-related Inflammatory Factor | -0.075 | 0.134 | 0.030 |
| Age | 0.187 | 0.415 | 0.301 |
| Sex | -0.199 | 0.037 | -0.081 |
| ApoE ε4 | -0.016 | 0.203 | 0.094 |
| Baseline MMSE score | -0.097 | 0.135 | 0.019 |
| Baseline Memory | 0.656 | 0.848 | 0.752 |
| Log Baseline White Matter Hyperintensities | -0.081 | 0.151 | 0.035 |
| Brain Parenchymal Fraction | 0.117 | 0.371 | 0.244 |
| Cholinesterase Inhibitor use | 0.050 | 0.375 | 0.212 |
| Memantine use | -0.145 | 0.122 | -0.012 |
| Anti-inflammatory medication use | -0.086 | 0.154 | 0.034 |
